# Supplementary material for: External validation of Finnish diabetes risk score (FINDRISC) and Latin American FINDRISC for screening of undiagnosed dysglycemia: Analysis in a Peruvian hospital health care workers sample
Source: PLoS One. 2024 Aug 7;19(8):e0299674. doi: 10.1371/journal.pone.0299674 (PMC11305586; doi:10.1371/journal.pone.0299674)
Supplement: S1 Table — (DOCX) [file pone.0299674.s001.docx]

**S1 Table. Scores of FINDRISC and LAFINDRISC**

|  |  | FINDRSC | LAFINDRISC |
| --- | --- | --- | --- |
| **Age (years)** | |  |  |
|  | <45 | 0 points | 0 points |
|  | 45-54 | 2 points | 2 points |
|  | 55-64 | 3 points | 3 points |
|  | ≥ 65 | 4 points | 4 points |
| **Body mass index (kg/m2)** | |  |  |
|  | <25 | 0 points | 0 points |
|  | 25 – 29.9 | 1 point | 1 point |
|  | ≥ 30 | 3 points | 3 points |
| **Waist circumference** | |  |  |
|  | M: <94 / F: <80 | 0 points |  |
|  | M: 94-101.9 / F: 80-87.9 | 3 points |  |
|  | M: ≥ 102 / F: ≥ 88 | 4 points |  |
| **Waist circumference (cm)** | |  |  |
|  | M: < 94 / F: <90 |  | 0 points |
|  | M: ≥ 94 / F: ≥ 90 |  | 4 points |
| **Regular med hypertension** | |  |  |
|  | No | 0 points | 0 points |
|  | Yes | 2 points | 2 points |
| **History of high glucosa** | |  |  |
|  | No | 0 points | 0 points |
|  | Yes | 5 points | 5 points |
| **Physical activity** | |  |  |
|  | Yes | 0 points | 0 points |
|  | No | 2 points | 2 points |
| **Fruits and vegetables** | |  |  |
|  | Every day | 0 points | 0 points |
|  | Not every day | 1 points | 1 points |
| **Diabetes in relatives** | |  |  |
|  | No | 0 points | 0 points |
|  | Yes, grandparents, cousings, uncle, aunt | 3 points | 3 points |
|  | Yes, parents, siblings, son, daughter | 5 points | 5 points |

M: male. F: female
