# Supplementary material for: External validation of Finnish diabetes risk score (FINDRISC) and Latin American FINDRISC for screening of undiagnosed dysglycemia: Analysis in a Peruvian hospital health care workers sample
Source: PLoS One. 2024 Aug 7;19(8):e0299674. doi: 10.1371/journal.pone.0299674 (PMC11305586; doi:10.1371/journal.pone.0299674)
Supplement: S2 Table — (DOCX) [file pone.0299674.s002.docx]

**S2 Table. Performance of LA-FINDRISC regarding different cut-off points**

| Cutpoint | Sensitivity | Specificity | Correctly Classified | LR+ | LR- | Youden Index |
| --- | --- | --- | --- | --- | --- | --- |
| ( >= 0 ) | 100.0% | 0.0% | 17.9% | 1.00 |  | 0.00 |
| ( >= 1 ) | 100.0% | 0.4% | 18.2% | 1.00 | 0.00 | 0.00 |
| ( >= 2 ) | 100.0% | 2.2% | 19.7% | 1.02 | 0.00 | 0.02 |
| ( >= 3 ) | 99.0% | 3.8% | 20.8% | 1.03 | 0.27 | 0.03 |
| ( >= 4 ) | 98.0% | 7.8% | 23.9% | 1.06 | 0.26 | 0.06 |
| ( >= 5 ) | 98.0% | 12.6% | 27.9% | 1.12 | 0.16 | 0.11 |
| ( >= 6 ) | 95.9% | 16.4% | 30.6% | 1.15 | 0.25 | 0.12 |
| ( >= 7 ) | 92.9% | 23.7% | 36.1% | 1.22 | 0.30 | 0.17 |
| ( >= 8 ) | 90.8% | 29.5% | 40.4% | 1.29 | 0.31 | 0.20 |
| ( >= 9 ) | 83.7% | 38.4% | 46.5% | 1.36 | 0.43 | 0.22 |
| ( >= 10 ) | 82.7% | 43.2% | 50.3% | 1.46 | 0.40 | 0.26 |
| **( >= 11 )** | **78.6%** | **51.7%** | **56.5%** | **1.63** | **0.41** | **0.30** |
| ( >= 12 ) | 69.4% | 59.7% | 61.4% | 1.72 | 0.51 | 0.29 |
| ( >= 13 ) | 61.2% | 67.9% | 66.7% | 1.90 | 0.57 | 0.29 |
| **( >= 14 )** | **58.2%** | **76.3%** | **73.0%** | **2.45** | **0.55** | **0.34** |
| ( >= 15 ) | 50.0% | 80.5% | 75.1% | 2.56 | 0.62 | 0.30 |
| ( >= 16 ) | 37.8% | 86.5% | 77.8% | 2.79 | 0.72 | 0.24 |
| ( >= 17 ) | 30.6% | 91.1% | 80.3% | 3.45 | 0.76 | 0.22 |
| ( >= 18 ) | 24.5% | 94.9% | 82.3% | 4.80 | 0.80 | 0.19 |
| ( >= 19 ) | 18.4% | 96.7% | 82.7% | 5.52 | 0.84 | 0.15 |
| ( >= 20 ) | 14.3% | 97.8% | 82.9% | 6.44 | 0.88 | 0.12 |
| ( >= 21 ) | 11.2% | 98.7% | 83.1% | 8.44 | 0.90 | 0.10 |
| ( >= 22 ) | 9.2% | 99.3% | 83.2% | 13.81 | 0.91 | 0.09 |
| ( >= 23 ) | 6.1% | 99.3% | 82.7% | 9.20 | 0.95 | 0.05 |
| ( >= 24 ) | 3.1% | 99.8% | 82.5% | 13.81 | 0.97 | 0.03 |
| ( >= 25 ) | 1.0% | 99.8% | 82.2% | 4.60 | 0.99 | 0.01 |
| ( > 25 ) | 0.0% | 100.0% | 82.2% |  | 1.00 | 0.00 |
