# Supplementary material for: External validation of Finnish diabetes risk score (FINDRISC) and Latin American FINDRISC for screening of undiagnosed dysglycemia: Analysis in a Peruvian hospital health care workers sample
Source: PLoS One. 2024 Aug 7;19(8):e0299674. doi: 10.1371/journal.pone.0299674 (PMC11305586; doi:10.1371/journal.pone.0299674)
Supplement: S3 Table — (DOCX) [file pone.0299674.s003.docx]

**S1 Table. Performance of original FINDRISC regarding different cut-off points**

| Cutpoint | Sensitivity | Specificity | Correctly Classified | LR+ | LR- | Youden Index |
| --- | --- | --- | --- | --- | --- | --- |
| ( >= 0 ) | 100.0% | 0.0% | 17.9% | 1 |  | 0.00 |
| ( >= 1 ) | 100.0% | 0.2% | 18.0% | 1.00 | 0.00 | 0.00 |
| ( >= 2 ) | 100.0% | 1.1% | 18.8% | 1.01 | 0.00 | 0.01 |
| ( >= 3 ) | 100.0% | 1.8% | 19.3% | 1.02 | 0.00 | 0.02 |
| ( >= 4 ) | 100.0% | 3.6% | 20.8% | 1.04 | 0.00 | 0.04 |
| ( >= 5 ) | 100.0% | 4.9% | 21.9% | 1.05 | 0.00 | 0.05 |
| ( >= 6 ) | 98.0% | 7.8% | 23.9% | 1.06 | 0.26 | 0.06 |
| ( >= 7 ) | 95.9% | 12.2% | 27.1% | 1.09 | 0.33 | 0.08 |
| ( >= 8 ) | 91.8% | 21.1% | 33.7% | 1.16 | 0.39 | 0.13 |
| ( >= 9 ) | 86.7% | 29.3% | 39.5% | 1.23 | 0.45 | 0.16 |
| ( >= 10 ) | 83.7% | 37.7% | 45.9% | 1.34 | 0.43 | 0.21 |
| ( >= 11 ) | 77.6% | 46.3% | 51.9% | 1.45 | 0.48 | 0.24 |
| **( >= 12 )** | **70.4%** | **53.9%** | **56.8%** | **1.53** | **0.55** | **0.24** |
| ( >= 13 ) | 62.2% | 62.5% | 62.5% | 1.66 | 0.60 | 0.25 |
| **( >= 14 )** | **59.2%** | **72.3%** | **70.0%** | **2.14** | **0.56** | **0.31** |
| ( >= 15 ) | 49.0% | 78.3% | 73.0% | 2.25 | 0.65 | 0.27 |
| ( >= 16 ) | 38.8% | 85.4% | 77.1% | 2.65 | 0.72 | 0.24 |
| ( >= 17 ) | 30.6% | 90.5% | 79.8% | 3.21 | 0.77 | 0.21 |
| ( >= 18 ) | 25.5% | 94.7% | 82.3% | 4.79 | 0.79 | 0.20 |
| ( >= 19 ) | 18.4% | 96.5% | 82.5% | 5.18 | 0.85 | 0.15 |
| ( >= 20 ) | 15.3% | 97.8% | 83.1% | 6.90 | 0.87 | 0.13 |
| ( >= 21 ) | 12.2% | 98.9% | 83.4% | 11.04 | 0.89 | 0.11 |
| ( >= 22 ) | 10.2% | 99.3% | 83.4% | 15.34 | 0.90 | 0.10 |
| ( >= 23 ) | 6.1% | 99.3% | 82.7% | 9.20 | 0.95 | 0.05 |
| ( >= 24 ) | 3.1% | 99.8% | 82.5% | 13.81 | 0.97 | 0.03 |
| ( >= 25 ) | 1.0% | 99.8% | 82.2% | 4.60 | 0.99 | 0.01 |
| ( > 25 ) | 0.0% | 100.0% | 82.2% |  | 1.00 | 0.00 |
